# Process Evaluation of Nurse-Led Online Self-Management Support for Family Caregivers to Deal With Behavior Changes of a Relative With Dementia (Part 1): Mixed Methods Study

**DOI:** 10.2196/13002

**Published:** 2019-10-11

**Authors:** Judith G Huis in het Veld, Iris F M van Asch, Bernadette M Willemse, Paul-Jeroen Verkade, Anne Margriet Pot, Marco M Blom, Rob B M Groot Zwaaftink, Anneke L Francke

**Affiliations:** 1 Amsterdam UMC, Vrije Universiteit Amsterdam, Department of Public and Occupational Health, Amsterdam Public Health Research Institute Amsterdam Netherlands; 2 Netherlands Institute of Mental Health and Addiction, Trimbos Institute Utrecht Netherlands; 3 The Geriant Foundation Region North of Amsterdam Netherlands; 4 World Health Organization Geneva Switzerland; 5 Dutch Alzheimer’s Society Amersfoort Netherlands; 6 Netherlands Institute for Health Services Research (NIVEL) Utrecht Netherlands

**Keywords:** dementia, internet, eHealth, caregiver

## Abstract

**Background:**

Coping with behavioral changes is a daily challenge for family caregivers in all phases of dementia, and assistance is needed for it. An online self-management support intervention was therefore developed and conducted involving the following elements: (1) email contact with a specialized dementia nurse, (2) online videos, and (3) e-bulletins containing information about behavior changes and how to manage them.

**Objective:**

The aim of this study was to understand (1) family caregivers’ actual use of various elements of the online self-management support, (2) family caregivers’ evaluation and satisfaction with the various elements, and (3) nurses’ usage and evaluations of the online support through the tailored email contacts.

**Methods:**

A mixed methods design was used in this process evaluation, combining quantitative and qualitative methods including analyses of dementia nurses’ registration forms, the number of clicks on online videos and e-bulletins, evaluation questions answered by family caregivers in a survey questionnaire, semistructured interviews with family caregivers and nurses, and analysis of the content of the email contacts.

**Results:**

The actual use of various elements of the online self-management support by family caregivers varied: 78% (21/27) of family caregivers had an email contact with the specialist nurse, 80% (43/54) of family caregivers clicked on an online video, and 37% (30/81) clicked on an e-bulletin. Family caregivers showed positive evaluations and satisfaction. The tailor-made approach in the personal email contacts in particular was valued by the family caregivers. Nurses’ evaluations about providing self-management support online were mixed as it was a relatively new task for them.

**Conclusions:**

An important insight is that not all participants made optimum use of the various elements of the intervention. Nurses also said that the email contacts were more often used to express feelings about coping with behavioral changes. More research is needed to investigate the reasons why people accept, adopt, and adhere to online interventions to reduce cases where they are not used and to back them up appropriately with tailored (online) information and advice for their personal situations.

## Introduction

### Background

Family caregivers of people with dementia often face many challenges in everyday life while caring for their relative [1], most prominently regarding changes in behavior of the person with dementia [2,3]. People with dementia may exhibit behavior that is dependent, aggressive, suspicious, apathetic, or indifferent, or night-time restlessness and masking behavior. Approximately 80% to 90% of people with dementia show behavior disturbances during the disease process [4], often distressing both the person with dementia and their family caregivers [3,5].

Coping with behavioral changes is a daily challenge for family caregivers in all phases of dementia [6]. These days, the term *self-management* is widely used when referring to managing consequences of a disease in daily life. Barlow et al [7] defined self-management as *the individual’s ability to manage the symptoms, treatment, physical and psychosocial consequences and lifestyle changes inherent in living with a chronic condition.* Self-management applies not only to the patient but also to family caregivers. Especially in dementia care, the person often becomes increasingly dependent on support from family caregivers. This is stressful for family caregivers, especially when coping with behavioral changes [5,6]. They use strategies to respond to behavioral changes by remaining calm or encouraging activities and distractions. Moreover, family caregivers have self-management strategies to manage their own caregiver stress and problems related to their relative’s dementia [5,8].

However, family caregivers might need assistance coping with this daily challenge. In particular, nurses are in the best position to help them because they develop a close partnership with individuals and their families throughout their lives [9]. This nurse-patient contact can also occur online [10] and might be especially useful for reaching family caregivers who are short of time because of providing care, have transportation difficulties or do not want to leave the person with dementia alone at home [11,12]. In addition to professional support online, family caregivers may also benefit from multicomponent online interventions that combine, for example, information and tailored caregiving strategies [13].

In this paper, we present a process evaluation of an online self-management support intervention addressing behavioral changes in dementia. The intervention consists of various online elements. The process evaluation was performed alongside a randomized controlled trial (RCT) [14]. The aim of the RCT was to explore (1) whether a major online self-management support intervention involving email contacts with a specialist dementia nurse in combination with online videos and e-bulletins is more effective than minor interventions not involving the email contacts with the nurse, and (2) if a medium intervention including videos and e-bulletins is more effective than a minor intervention including e-bulletins only. The results showed no statistically significant effects on family caregivers’ self-efficacy for the major and medium online self-management support interventions compared with the minor intervention [15].

It is important to carry out a process evaluation alongside RCTs to allow effects (or the lack thereof) to be interpreted. It enables researchers to understand whether and how interventions are used, and how interventions are being evaluated by the people involved. Process evaluations alongside RCTs are even more important when evaluating online interventions because these studies are complicated, given the high numbers of nonadherent participants compared with face-to-face interventions [16,17].

### Objective

The overall objective of the process evaluation was to get an idea of the actual usage and evaluations of the intervention components. Related subobjectives were to understand (1) actual usage by family caregivers of the various elements of the online support, (2) family caregivers’ evaluation of and satisfaction with the various elements, and (3) nurses’ usage and evaluations of the online support through the tailored email contacts.

## Methods

### Design

The process evaluation had a mixed methods design in which quantitative and qualitative methods were combined, and various sources were used (see the *Data Collection* section). The process evaluation was performed alongside the RCT involving 3 intervention arms (see the section *Interventions: Content and Development Trajectory*). Following the definition given by the Medical Research Council, the aim of this process evaluation was to understand the functioning of a complex intervention consisting of multiple components [18]. The design of the RCT is described elsewhere [14].

### Participants

Family caregivers as well as specialized dementia nurses participated in the process evaluation.

Inclusion criteria for family caregivers were the same as the criteria used in the RCT: family caregivers aged at least 18 years, who were a partner or relative of a person diagnosed with dementia who is living at home, having contact at least weekly with the person with dementia, with internet access and who provided online consent [14]. In total, 81 family caregivers participated in the RCT (major [n=27], medium [n=27], or minor [n=27] intervention arms).

Inclusion criteria for the specialized dementia nurses were: (1) a Bachelor’s or Master’s degree in nursing, and (2) advanced training in dementia care. In total, 4 nurses participated.

### Interventions: Content and Development

Family caregivers were randomly allocated to 1 (major), 2 (medium), or 3 (minor) intervention arms.

1. The major intervention arm was the most comprehensive and consisted of the following 3 elements:
  The first element consisted of email contacts with a specialized dementia nurse thrice. In the email contacts, the nurse helped family caregivers online to manage behavioral changes, guided by an intervention protocol developed by the project team members (JGH, ALF, PJV, and IvA), in consultation with the nurses themselves. The Dutch protocol (available on request from the first author) was structured using the steps of the 5A model of self-management support [19,20] and Kitwood’s person-centered care theory [21]. The 5A model comprises the following 5 steps: assessing; advising; agreeing on goals; assisting in anticipating barriers and developing a specific action plan; and arranging follow-up [19,20,22,23].
  Another element was providing online videos on how to manage the relative’s behavior changes and how to improve self-efficacy in managing this behavior. There were 6 videos dealing with different common types of behavior changes: dependent behavior, suspicious behavior, aggressive behavior, apathy or indifference, restlessness at night, and masking behavior. Each video had the same structure, starting with possible causes and related solutions for responding or coping with the specific behavior, and ending by emphasizing that it is important that family caregivers take good care of themselves. Family caregivers could choose how many videos they watched depending on their personal needs and the behavioral changes encountered in their relative with dementia. The videos (along with the e-bulletins mentioned below—see element c) were developed by the coauthors BMW, IvA, and AMP, in close collaboration with colleagues from the Trimbos Institute and the Dutch Alzheimer’s Society, family caregivers of people with dementia and other experts. In the first step of the development process, a desk search was performed to obtain an impression of what is known in the literature about methods of influencing behavior approached from a person-centered perspective [21] and how family caregivers experience different kinds of behavioral changes in their relative with dementia. Experts also provided input for various aspects of the videos (eg, cognitive behavioral therapy principles, persuasive communication, modeling, and active learning). Video scripts and pilot videos were tested by family caregivers at several points during development. The videos are available on https://dementie.nl/online-training.
  Providing e-bulletins containing practical information about various types of behavioral changes and how to manage them was the third element. The same behavior changes were covered in the e-bulletins as in the videos. The e-bulletins included assignments that were designed to help caregivers interpret the generic information in the context of their own situation, to reflect on what might be causing the behavior changes, how they would like to cope with the behavior, and how they would like to respond. During the development process, the e-bulletins were tested together with the online videos. They have the same theoretical basis as the videos, and the people involved in the development of the videos were also involved in developing the e-bulletins.
2. The medium intervention, consisting only of the online videos and e-bulletins (elements b and c above);
3. The minor intervention, consisting only of the e-bulletins (element c).

### Data Collection

A schematic overview of the data collection methods used is given in Table 1. In some parts of the process evaluation, the sample concerned all family caregivers participating in the RCT (n=81), whereas in other parts of the process evaluation, only subsamples participated.

As can be seen in the second column of Table 1, quantitative data involved nurses’ records of the number of times personal email contacts occurred per family member, clicks on links to the online videos and e-bulletins, and evaluation questions answered by family caregivers in a questionnaire. The evaluation questions were part of the questionnaire used at the end of the RCT [15].

As shown in the third column of Table 1, the qualitative data were collected in semistructured interviews with family caregivers.

In the last questionnaire used at the end of the RCT, family caregivers were asked if they would like to take part in such an interview. In total, 41 family caregivers were willing to participate. Of these, 12 were purposively recruited with a spread of intervention arms and background characteristics (eg, gender, age, and living with or separately from the relative with dementia). They were sent an information letter by email and were asked to give their consent by email if they were willing to be interviewed. All interviews were conducted by telephone by one of the coauthors.

Semistructured interviews were also conducted with the 4 specialized dementia nurses who provided the personalized email contacts with the family caregivers. The topic list addressed how the nurses evaluated their support in the personal tailored email contacts. All interviews with the nurses were carried out by one researcher (IvA). Three interviews were conducted by telephone; one interview took place at the Trimbos Institute.

Finally, the content of email contacts was analyzed regarding family caregivers’ request for help, referral to the online videos and nurses’ use of the intervention protocol based on the 5A model.

**Table 1:** Data collection methods (quantitative and qualitative) used for each research question.

Research aims	Quantitative data	Qualitative data	Data collection period
To understand the actual use of family caregivers of the elements of the self-management support	Recording the actual use of personal email contact with nurse by 27 family caregivers	—^a^	March to August 2017
	Clicks on the video links by 54 family caregivers	—	March to August 2017
	Clicks on the e-bulletin links by 81 family caregivers	—	March to August 2017
To understand family caregivers’ evaluation and satisfaction with the various elements of the online self-management support interventions	Evaluation questions in a survey with Likert scale, send to 81 family caregivers	—	March to August 2017
	—	Semistructured interviews with 12 family caregivers	July and August 2017
To understand nurses’ usage and evaluations of the online support through tailored email contacts	—	Semistructured interviews with 4 nurses	September 2017
	—	Analysis of the content of email contacts between 27 family caregivers and nurse	March to August 2017

^a^Not applicable.

### Data Analysis

#### Quantitative Data

Records and clicks on links were descriptively analyzed using Microsoft Excel (version 2010). The evaluation questions in the questionnaire were analyzed descriptively (frequencies and percentages) using SPSS software (IBM Corporation).

#### Qualitative Data

The transcribed verbatim audio-recorded interviews were analyzed independently by 2 researchers (JGH and IvA) using the principles of thematic analysis [24]. First, the researchers repeatedly read the data and looked for meanings and patterns in the data. Second, an initial list of codes was generated about what was in the data and what was interesting for the research questions. Third, the various codes were sorted into potential themes and then fourth, refined so that data within the themes fitted together meaningfully. Fifth, the themes were further refined by analyzing the data within the themes. Finally, once there was a set of fully detailed themes, the final analyses were written down [24]. Coding and interpretation of the codes were discussed at several moments in the analysis process by the researchers to reach consensus and to refine the analyses. In addition, interim and final analyses were also discussed with other coauthors. Furthermore, member checking was performed by discussing interim and final analyses with one of the nurses who was involved in the email contacts (PJV).

The content of email contacts between 27 family caregivers and the nurses was analyzed by 1 researcher (JGH) looking at their request for help, referral to the online videos and the use of the 5A model. A second reviewer (ALF) screened a random selection (email contacts of 3 family caregivers).

### Ethical Considerations

The Medical Ethics Committee of the Vrije University Medical Center approved this study (reference 2016.559). All participating family caregivers and dementia nurses gave informed consent. All data were stored according to the rules of the Dutch Data Protection Act.

## Results

The collected quantitative and qualitative data are categorized based on the 3 different elements of the intervention: email contacts with nurses, online videos, and e-bulletins.

### Family Caregivers’ Usage of Email Contacts With Nurses

A total of 27 family caregivers were assigned to the major intervention arm, meaning that they had the opportunity to have personal email contact with a dementia nurse in addition to the videos and e-bulletins. Of the 27 family caregivers, 21 (78%) actually made use of the opportunity. Almost half of the family caregivers (13/27, 48%) had email contacts thrice, 4 had twice (15%), and another 4 (15%) had once (Table 2).

**Table 2:** Data from the recording form for personal email contacts kept by the nurses.

Personal email contacts		Value
**Family caregivers in the major intervention arm, n**		27
	Had email correspondence thrice	13
	Had email correspondence twice	4
	Had email correspondence once	4
	No email correspondence	6
Total number of times the email contacts occurred		51
Time spent per email contact by nurses (min), mean (range)		35 (20-55)

### Family Caregivers’ Evaluation and Satisfaction With Email Contacts With Nurses

A total of 27 family caregivers assigned to the major intervention arm were asked to complete evaluation questions about the email contacts (Table 2, second column). A total of 16 family caregivers completed the evaluation questions and had email contact with a nurse. The majority (12/16; 75%) valued the personal email contacts with the nurses in addition to the videos and e-bulletins. The nurses’ explanation and advice given in the email contacts were clear for most family caregivers (12/16, 75%), and more than half of them (9/16, 56%) said they could immediately use the nurses’ advice in managing the behavior of their relative with dementia (Table 3).

A total of 4 family caregivers in the major intervention arm were interviewed about how they evaluated the personal email contacts with the nurse. They stated that they got the most out of these contacts, compared with online videos and e-bulletins, because of the personal aspect. They appreciated that they were given the opportunity to reflect with the nurse on how they were dealing with their relative’s situation, which made them aware that they had to take a step back in some situations. They also liked the tips and ideas that the nurses gave them about how to act in their situation. In addition, they said that it was good to get confirmation that you were doing it correctly:

Now like I said: you talk. At least you are then communicating with somebody [the nurse] who understands what it's about. You don't have to keep on reinventing the wheel then, in fact. You can just say, well, I'm coming up against this and that. Oh—watch out for this, watch out for that. That's simply very pleasant, I reckon.

Participant 10

One family caregiver said that she did not use the email contacts. The reasons were not only the lack of time but also that the counselling by email was not attractive because you then have to put your emotions and questions on paper. That was a barrier for this family caregiver, who also said that the barrier would have been much lower if the counselling had been by phone.

**Table 3:** Evaluation questions on Likert scales.

Survey questions		Major (n^a^=16), n (%)	Medium (n=21), n (%)	Minor (n=15), n (%)
**The personal email correspondence with the nurse added value to the video and e-bulletins**				
	Completely agree/agree	12 (75)	—^b^	—
	Neutral	2 (12)	—	—
	Disagree/completely disagree	2 (12)	—	—
**The nurse’s explanation and advice were clear**				
	Completely agree/agree	12 (75)	—	—
	Neutral	3 (19)	—	—
	Disagree/completely disagree	1 (6)	—	—
**I was able to use the advice of the nurses immediately in managing the behavior of my relative with dementia**				
	Completely agree/agree	9 (56)	—	—
	Neutral	6 (37)	—	—
	Disagree/completely disagree	1 (6)	—	—
**The videos and e-bulletins fitted my situation**				
	Completely agree/agree	10 (62)	9 (43)	—
	Neutral	6 (37)	11 (52)	—
	Disagree/completely disagree	0 (0)	1 (5)	—
**The videos and e-bulletins helped me to manage the behavior of my relative with dementia**				
	Completely agree/agree	11 (69)	10 (48)	—
	Neutral	5 (31)	10 (48)	—
	Disagree/completely disagree	0 (0)	1 (5)	—
**In addition to videos and e-bulletins, I would have liked to receive extra support from a nurse by email**				
	Completely agree/agree	—	6 (29)	—
	Neutral	—	13 (62)	—
	Disagree/completely disagree	—	2 (10)	—
**The e-bulletins fitted my situation**				
	Completely agree/agree	—	—	7 (47)
	Neutral	—	—	6 (40)
	Disagree/completely disagree	—	—	2 (13)
**The e-bulletins helped me to manage the behavior of my relative with dementia**				
	Completely agree/agree	—	—	8 (53)
	Neutral	—	—	5 (33)
	Disagree/completely disagree	—	—	2 (13)
**In addition to the e-bulletins, I would have liked to receive extra support from a nurse by email**				
	Completely agree/agree	—	—	6 (40)
	Neutral	—	—	6 (40)
	Disagree/completely disagree	—	—	3 (20)

^a^Family caregivers who completed the evaluation questions.

^b^Data not applicable.

### Family Caregivers’ Usage of Online Videos

In total, 54 family caregivers (27 in the major intervention arm and 27 in the medium intervention arm) had access to 6 videos about how to manage behavioral changes in their relative with dementia. Of them, 43 (80%) clicked at least 1 video. Clicks on the videos are listed in Table 4.

**Table 4:** Clicks on the links to the videos and e-bulletins.

Clicks on videos and e-bulletins		n (%)
**Total number of family caregivers who clicked videos**		43 (80)
	Family caregivers in the major intervention arm who watched at least one video (n=27)	22 (81)
	Family caregivers in the medium intervention arm who watched at least one video (n=27)	21 (78)
**Total clicks on e-bulletins**		30 (37)
	Family caregivers in the major intervention arm who watched at least one e-bulletin (n=27)	5 (19)
	Family caregivers in the medium intervention arm who watched at least one e-bulletin (n=27)	7 (26)
	Family caregivers in the minor intervention arm who watched at least one e-bulletin (n=27)	18 (67)

### Family Caregivers’ Evaluation and Satisfaction With the Videos and E-Bulletins

A total of 54 family caregivers were asked to complete evaluation questions about the videos (Table 3, second column). A total of 37 caregivers watched at least 1 video (16 in the major intervention arm and 21 in the medium intervention arm). Half of them (both major and medium arms, 19/37, 51%) said that the videos and e-bulletins fitted their personal situation, and more than half stated that they helped them to better manage with the behavior of the person with dementia (21/37, 57%).

In total, 9 family caregivers who had access to the videos (4 in the major intervention arm and 5 in the medium intervention arm) were interviewed about how they evaluated the videos. They said that they thought the videos were well-structured and pleasant to watch. They also found the content clear and useful. The tips given in them were reckoned to be useful; watching the videos gave them new ideas for coping with the behavioral changes in their relative:

Well, because it's important for you to have a clear picture as well. It's useful to know what I ought to be doing. That really does help quite a bit. Otherwise there's a lot of conflict and so forth, instance defiance or whatever—quite a lot. It lets you know how to tackle the situation: let's put it like that.

Participant 5

Some of the family caregivers found the content and the stories of other family caregivers recognizable and helpful. Others said they did not relate to much that was in the videos because there was no change in behavior in their situation or the behavior was expressed differently. They said that this meant the videos were less useful to them:

With my husband, it was mostly about the aggression and waking up at night and that wasn't something I really saw in the video or in the text. And that was what I find so typical. There were a few bits in that I recognized, but I didn't get the feeling that the situation really fitted in very well with ours.

Participant 4

One family caregiver also remarked that the videos and the e-bulletins were suitable primarily in the early phases of dementia; another said that the information was too sketchy for family-based caregivers dealing with dementia in its later stages.

### Family Caregivers’ Usage of E-Bulletins

All family caregivers (n=81) had access to the e-bulletins (through a link). The e-bulletins contained practical information about various types of behavioral changes and tasks to help reflect on their possible causes and how to influence and cope with them. In total, 30 family caregivers out of 81 (37%) clicked at least 1 e-bulletin. In the minor intervention arm, the percentage who clicked the e-bulletins was the highest (18/27, 67%; Table 3).

### Family Caregivers’ Evaluation and Satisfaction With E-Bulletins

Of 27 family caregivers in the minor intervention arm, 15 (56%) answered the evaluation questions (Table 3, fourth column). Almost half (7/15, 46%) said that the e-bulletins fitted their situations and that the e-bulletins helped them to manage behavioral changes in the person with dementia (8/15, 53%).

In total, 12 family caregivers (4 in the major intervention arm, 5 in the medium intervention arm and 3 in the minor intervention arm) were interviewed on how they evaluated the e-bulletins. A number of family caregivers stated that the information in the e-bulletins was clear and recognizable as well as being helpful to read again. Some also said that one of the benefits was that there was one e-bulletin of each type of behavior. Conversely, others felt that the content of the e-bulletins was not always recognizable and that they were unable to translate it well to their own situations. One family caregiver said that the e-bulletins were not concrete enough, and she also perceived the e-bulletins as a bit patronizing at times. On the other hand, this family caregiver also said that this might be just a personal opinion.

Some of the group who had also seen the video felt that the e-bulletin was a good addition to the videos, whereas others set more store by it because information from the videos was enough for them.

The family caregivers who only received the e-bulletins mostly thought they were informative, although one family caregiver said that information did not help in her situation. Others said that the information meant they were more aware of what they could come up against and that it put a different perspective on the behavior for them. Moreover, understanding the behavior better because of the information from the e-bulletins let them be more patient in dealing with the behavior:

Explaining the behavior and how you have to respond to it, right? Most of the time you have to count to ten first or—as I always say—sometimes a hundred. Like that.

Participant 3

The family caregivers would recommend the e-bulletins to others. One of them advised distributing this information among professionals too, having noted that they do not always know enough about behavioral changes.

### Nurses’ Evaluation and Satisfaction With Providing Tailored Email Contacts

Four specialized dementia nurses provided online self-management support via email. In total, the nurses had email contacts with family caregivers 51 times. The time spent by the nurses varied from 20 min to 55 min (mean 35 min) per email contact (Table 1).

Semistructured interviews were held with 4 nurses to get an idea of their use and evaluations of providing online self-management support. Categorization resulted in 4 themes: background characteristics and expectations of family caregivers, evaluation of the online assistance, evaluation of the intervention protocol with the 5A model, number of times the email contacts, and the perceived effect.

### Background Characteristics and Expectations of Family Caregivers

Two specialist nurses said that the family caregivers had partners in an advanced stage of dementia. One nurse said that she got the impression that the family caregivers were overloaded. Moreover, the nurses noted that some of the caregivers had one or more people helping them and were deliberately busy collecting information about the condition. In addition, a nurse said that the family caregivers were not aware that they were also tackling their situations too.

In terms of the expectations of the family caregivers, the majority of the nurses had the impression that family caregivers were looking for a release valve and a listening ear. A number of the caregivers needed concrete ideas about how to deal with behavioral changes in their relative. One nurse also said that she noticed that she was being asked questions about case management, for instance about coordinating care for the relative.

### Evaluation of the Online Assistance

The nurses said that there were pros and cons to giving online assistance. One nurse said that putting the situation down on paper was one of the benefits of online counselling because the family caregivers then got a better picture of the severity, and the situation would sink in more quickly:

Yes [...] because the family caregivers are e-mailing and putting things into words, the seriousness of the problem is made a bit clearer, I reckon. I get that idea quite strongly. Putting it on paper can in fact point out the severity—almost as if they're saying they can't cope any more. Yes, that does help. It paints a picture of the changed behavior, and shows that action is needed as well.

Nurse 1

They also felt it was an advantage that you can ask encouraging questions, but the nurse wondered whether this matched the family caregivers' expectations of this online assistance.

Giving online counselling was also felt to be *awkward* because you cannot look anyone in the eye, and it is then more difficult to assess the situation. They found it tricky to get the right tone for approaching the family caregiver. As the counselling was online, the nurse did not know if the advice had been understood by the family caregiver. If the caregiver no longer responded, the nurse did not know if they had said something wrong or if there was another, unrelated reason.

Another nurse said that online assistance is suitable for practical questions, but that you need more time and need to know more in the role of health care provider if it is about people being overburdened or about changed behavior. Another nurse believed that it became easier as you did it more often. A certain amount of practice is needed if this counselling is to be provided properly.

### Evaluation of the Intervention Protocol With the 5A Model

One nurse said that the 5A model could help a lot in the online counselling but that the nurses had difficulties with the application of the model. The link between the video content and the 5A model was also unclear. The reason was that they had a feeling that the family caregivers needed other assistance, for example, providing a listening ear. The videos and email counselling focused on coping with the changed behavior, but the nurses noticed that the family caregivers had more of a need to talk about things. Getting them to talk about the behavioral changes and think about them felt like the nurses were pushing.

### Number of Times Email Contacts Occurred

Opinions varied as to whether the number of times the email contacts occurred was sufficient. Two of the nurses said that it was enough. One nurse did state as a condition that the contacts should then only focus on the behavioral changes and not on other questions and advice. Another nurse doubted whether contacts occurring 3 times was enough to have an effect. The emails from the family caregivers contained a lot of information, not only about to change behavior but also about the other problems involved. Another nurse said she got the impression that family caregivers enjoyed watching the videos but did not think that they actually wanted to do anything as result.

### The Perceived Effect

Most of the nurses said that their assistance meant that family caregivers could get things off their chest or that the family caregivers felt they had been listened to. One nurse said that it was a help that the family caregivers had taken a moment to think about the behavioral changes in their relative with dementia. She also thought that the tips she had given about how to make thorny subjects open to discussion had helped. According to one nurse, effective elements were the attention paid to the personal situations of the family caregivers and being able to reflect on them together. This nurse was also able to give the family caregivers tips about other forms of assistance. Another nurse believed that the email contacts had helped the family caregivers translate what was happening in the videos to their own situations. In the case of one family caregiver, a nurse had the impression that the counselling had no effect because the person in question was already so overburdened that email contact was too much. Another nurse did not believe that it had given the family caregivers a better picture of behavioral changes because the nurses did not have the right skills for online counselling and because the need for assistance among family caregivers was so diverse.

### Analysis of the Content of Email Contacts Between Family Caregiver and Nurse

Of 27 family caregivers (78%) 21 had email contact with a nurse. As data were missing for 2 family caregivers, email contacts from 19 family caregivers were analyzed. Of the 19 family caregivers, 11 (58%) did not express an explicit goal in the email contacts. In 15 cases (79%), the content of the emails was about behavioral changes in their relative with dementia. A total of 4 family caregivers (21%) also discussed about caregiving stress. A total of 6 (32%) family caregivers discussed other caregiving issues not related to behavioral changes. In 5 cases (26%), the nurse referred to the online videos in the email contacts (Table 5).

The first step in the 5A model (*assessing*) was used by the nurses in all email contacts. The second step (*advising*) was used in about half of the cases. The other steps of the 5A model (*agreeing on goals*, *assisting in anticipating barriers and developing a specific action plan*, and *arranging follow-up*) hardly occurred at all in the email contacts.

**Table 5:** Content of all email contacts between family caregivers and a nurse (n=19).

Email content		n (%)
**Explicitly formulated request for help**		
	Yes	8 (42)
	No	11 (58)
**Content discussed in 1 or more emails**		
	Behavioral changes	15 (79)
	Managing caregiver stress	4 (21)
	Other caregiving issues (other than behavioral changes of the relative with dementia)	6 (32)
**A link to the online videos**		
	Yes	5 (26)
	No	14 (74)

## Discussion

### Principal Findings

Through this process evaluation, we aimed to gain an idea of (1) actual use by family caregivers of the various elements of online self-management support, (2) family caregivers’ evaluation and satisfaction with the various elements, and (3) nurses’ usage and evaluations of the online support through the tailored email contacts. This process evaluation was performed alongside an RCT [14] in which the effectiveness was studied of an online self-management support intervention involving tailored email contacts with a specialized dementia nurse combined with online videos and e-bulletins. Contrary to our expectations, no statistical evidence was found for the major and medium online self-management interventions compared with minor intervention (involving e-bulletins only) on family caregivers’ self-efficacy [15]. Although no effects were found, this evaluation noted that family caregivers valued the email contacts with the specialist nurse. They mentioned that receiving confirmation from a professional that they were doing the right thing was really important to them. Previous studies also found that being acknowledged by professionals and peers for the everyday care they provided is extremely important for family caregivers in helping them cope with daily challenges [8,25]. They also felt that the email contacts offered added value above the videos and e-bulletins. Family caregivers who received the videos and e-bulletins mentioned difficulties in translating the information and advice to their own situations. It could therefore be suggested that an online personal approach is needed to acknowledge the highly complex situation of family caregivers and subsequently assist them by providing tailored online information and advice for their personal situations.

This process evaluation also suggests possible explanations for the unexpected results in the RCT by understanding how the intervention was used and was evaluated by the people involved. First, this process evaluation showed variation in the extent that family caregivers made use of the various elements of the online self-management support. 78% of family caregivers had an email contact with a nurse (21/27), 80% watched 1 or more online videos (43/54), and 37% clicked an e-bulletin (30/81). The distinction between the 3 intervention arms consequently becomes less, making it difficult to demonstrate effects [17]. Nonuse of an intervention is a methodological known difficulty in online trials and may explain why interventions fail to show a measurable effect for the intervention [17,26,27]. For electronic health (eHealth) interventions to present an effect, they need to be accepted and used in the intended way to benefit the participants the most [28]. However, improving the use of eHealth interventions is complex, and more insights are needed for investigating the reasons why people accept, adopt, and adhere to eHealth interventions so that their behavior can be influenced [28].

Second, according to the nurses, the participants involved in the email contacts were mainly family caregivers who availed the services of 1 or more health professionals and were highly engaged in collecting information about dementia. This group would then already have information and advice on how to cope with behavioral changes, which might explain why family caregivers wanted to share their stories and express their feelings instead of finding other ways to self-manage the behavioral changes of their relative with dementia. For future research, it is important to determine which family caregivers will benefit most from what type of support. This would provide insight that can be used to provide the intervention in a more cost-effective way. This, for example, means that nurses’ support can be provided to the people who are likely to benefit most.

Another possible explanation for finding no statistical evidence for the benefits of email contact between family caregivers and nurses (combined with videos and e-bulletins) may be how the intervention was carried out. In many cases, only the first two A’s (*assessing* and *advising*) were completed. Using the 5A model turned out to be difficult as it was new to the nurses. Previous studies’ results were comparable, as the last 2 A’s (*assist* and *arrange*) seem to be delivered least often by nurses [19,29,30]. However, those components are most important for producing meaningful and lasting behavioral changes [19]. Future research therefore needs to investigate how all steps of the 5A model could be performed online.

When providing online support, the dosage of online intervention should also be considered. It is for instance striking that only a few (37%, 30/81) family caregivers clicked e-bulletins. This could be explained by the fact that not everything that is offered will also be used. This may be illustrated by the low usages rates of the e-bulletins by family caregivers who also had email contact with a nurse and access to the online videos. This indicates that informal caregivers do not stick to the intervention but decide for themselves what care is needed and fits their unique situation. Tailored information and advice should therefore be offered in a way that is geared to family caregivers’ needs [31]. This could include a differentiated offer of support instead of offering multiple kinds of support. This enables family caregivers get help that is based on their needs, self-management abilities, and home situations.

### Strengths and Limitations

The mixed methods design combining quantitative and qualitative data enabled better understanding of how online self-management support interventions were used and evaluated by both the family caregivers and dementia nurses involved. Using telephone interviews let family caregivers participate without extra traveling time for the family caregiver or researcher. The information gathered can be used to develop online self-management support further for families facing dementia. Furthermore, the validity of the results was enhanced by combining quantitative and qualitative data [32]. However, findings of this study need to be considered within the context of a number of methodological limitations. First, tracked usage data were measured in clicks that represent page views. People who click a link do not however necessarily watch the whole online video or read the e-bulletin. The numbers found could therefore overestimate family caregivers’ utilization of an intervention component. Click data should therefore be seen in combination with other evaluation methods [33]. Second, no data were collected for the 6 family caregivers who did not use the email contacts. Barriers preventing family caregivers from making email contact with a nurse could therefore potentially have not been detected.

### Conclusions

There was a variation in the extent to which family caregivers utilized the various elements of online self-management support. They valued the tailor-made approach in the email contacts. According to the nurses involved, online personal email contacts was mostly used to express feelings concerning coping with changing behavior. Nurses’ usage and evaluations of providing self-management support online were mixed, as it is a relatively new task for nurses. More research is needed to investigate the reasons why people accept, adopt, and adhere to online interventions to reduce nonuse and to support them appropriately by providing tailored (online) information and advice for their personal situations.

## Abbreviations

eHealth: electronic health
RCT: randomized controlled trial
